# Tissue Chips and Microphysiological Systems for Disease Modeling and Drug Testing

**DOI:** 10.3390/mi12020139

**Published:** 2021-01-28

**Authors:** Leslie Donoghue, Khanh T. Nguyen, Caleb Graham, Palaniappan Sethu

**Affiliations:** 1 Division of Cardiovascular Disease, University of Alabama at Birmingham, Birmingham, AL 35233, USA; lesdon@uab.edu (L.D.); ntkhanh@uab.edu (K.T.N.); cgraha5@uab.edu (C.G.); 2 Department of Biomedical Engineering, University of Alabama at Birmingham, Birmingham, AL 35294, USA

**Keywords:** tissue chips, microphysiological systems, microfluidics, organ-on-a-chip, tissue-on-a-chip, body-on-a-chip

## Abstract

Tissue chips (TCs) and microphysiological systems (MPSs) that incorporate human cells are novel platforms to model disease and screen drugs and provide an alternative to traditional animal studies. This review highlights the basic definitions of TCs and MPSs, examines four major organs/tissues, identifies critical parameters for organization and function (tissue organization, blood flow, and physical stresses), reviews current microfluidic approaches to recreate tissues, and discusses current shortcomings and future directions for the development and application of these technologies. The organs emphasized are those involved in the metabolism or excretion of drugs (hepatic and renal systems) and organs sensitive to drug toxicity (cardiovascular system). This article examines the microfluidic/microfabrication approaches for each organ individually and identifies specific examples of TCs. This review will provide an excellent starting point for understanding, designing, and constructing novel TCs for possible integration within MPS.

## 1. Introduction

For decades, our understanding of essential cellular functions and how they relate to human health has relied on model systems that investigate the molecular basis of normal physiology and pathophysiology. Cellular-level models provide a high degree of biological specificity that permits the study of specific cellular signaling pathways in the absence of noise or crosstalk due to interactions among other tissues and organs. However, the physiological relevance of cellular-level models is low, as they fail to recreate the systemic interactions seen in whole organisms. For this reason, while the identification of therapeutic targets typically begins at the cellular level, drugs require subsequent pre-clinical validation in higher-level animal models before progressing to human studies. The cost and time associated with obtaining regulatory approval following pre-clinical studies and clinical trials for a single drug are upwards of USD 2.55 billion and between 10–15 years, respectively [1,2,3]. A significant contributor to this expensive and time-consuming drug discovery pathway is the reliance on animal models, which oftentimes are not accurate predictors of the efficacy and toxicity of drugs in humans. Nearly 30% of drugs deemed safe in animal studies are toxic to humans, and around 60% of drugs that are effective in animals provide no discernible benefit to humans [4]. These discrepancies are due, in large part, to interspecies differences in the activities of drug-metabolizing enzymes. Thus, there is a critical need for alternatives to animal models that closely replicate human physiology to predict the efficacy, safety, bioavailability, and toxicity of candidate therapeutics.

A potential solution for increasing the predictive power of pre-clinical studies is the use of in vitro model systems constructed with human cells. However, such model systems’ clinical relevance relies on their ability to accurately mimic human physiology and function. Breakthroughs in developmental and stem cell biology have resulted in the availability of human induced pluripotent stem cells (hiPSCs), representing renewable and patient-specific source of cells for constructing complex, multicellular in vitro models. Recent advancements in tissue engineering, biomaterial science, three-dimensional (3D) fabrication techniques, and microfluidic technologies have enabled new methods for creating 3D tissue constructs or tissue chips (TCs). TCs, also frequently referred to as organ chips (OCs), replicate essential aspects of human organ structure and function, and their development has received strong support from federal funding agencies. In 2010, the National Institutes of Health (NIH) Common Fund, in collaboration with the Food and Drug Administration (FDA), started the Regulatory Science Program to make medical product development and evaluation more efficient. As part of this program, the first TC project, which sought to develop a heart-lung model to test the safety and efficacy of drugs, was funded [4]. This initiative was further strengthened via additional collaborations with the Defense Advanced Research Projects Agency (DARPA), leading to a coordinated effort to launch the “tissue chips for Drug Screening” program in 2012. More recently, their application has broadened to a multitude of disciplines. Researchers have since developed microphysiological systems (MPSs), also known as multi-organ tissue chips (MOTCs), established Tissue Chip Testing Centers, to validate their efficacy for disease modeling, and have sent TCs to the International Space Station U.S. National Laboratory to study the effects of microgravity on human cells [5]. (To avoid confusion, the terms “tissue chips” and “microphysiological systems” will be used exclusively throughout the rest of this review, except in cases where the original authors used the alternate terminology.)

**Figure 1:**
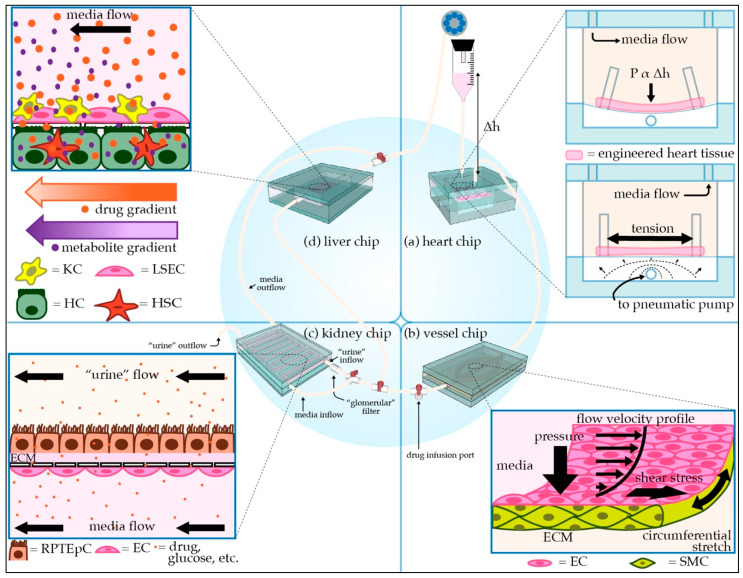
Schematic of modular microphysiological system. (**a**) Three-dimensional heart chip with cardiomyocytes (CMs) and stromal cells suspended in hydrogel between posts. The chip mimics the pressure-volume changes seen in the left ventricle. The “diastolic filling pressure,” which is directly proportional to the fluid reservoir’s height, pushes the flexible polydimethylsiloxane (PDMS) membrane downward, stretching the cardiac fiber. A pneumatic pump then generates “systolic pressure” in the lower air-filled chamber, returning the membrane and cardiac fiber to the baseline stretch. (**b**) Perfusable 3D vessel chip with microvessels composed of endothelial cells (ECs) and smooth muscle cells (SMCs) surrounded by an extracellular matrix (ECM), with key tunable parameters indicated. (**c**) Three-dimensional kidney chip mimicking the proximal tubule and adjacent peritubular capillary. To mimic the proximal tubule, renal proximal tubule epithelial cells (RPTEpCs) are cultured upon a bed of ECM. RPTEpCs have a prominent brush border, as they would in vivo. The underlying porous membrane recapitulates the selective barrier function of the tubule wall. In addition to the upstream drug infusion port, there is a valve allowing for media to bypass the kidney chip, as well as a valve splitting the kidney chip media inflow. One inflow branch passes through a “glomerular” filter and enters the proximal tubule chamber as “urine.” The remaining unfiltered media flows into the bottom vascular chamber, the superior aspect of which is lined with ECs. Other applications of this chip include modeling transport phenomena related to drugs or other key molecules. (**d**) Liver chip with liver sinusoidal endothelial cells (LSECs) and Kupffer cells (KCs) lining the “sinusoid,” a porous membrane mimicking the perisinusoidal space, and hepatocytes (HCs) and hepatic stellate cells (HSCs) cultured below the membrane. HCs have microvilli projecting towards the “perisinusoidal space,” as they would in vivo.

With TCs and MPSs (Figure 1) poised to become integral components of the drug discovery and regulatory process, the working definitions of the terms “tissue chips” and “microphysiological systems” need to be established. “tissue chips” can be defined as “engineered in vitro devices that can be used to model both structure and function of working units in the body, including organs such as the brain, heart, lungs, liver, gut, pancreas, and kidneys and tissues such as skeletal muscle, adipose tissue, and bone.” Typically, such models utilize cells (preferably of human origin, to avoid interspecies differences), extracellular matrix (ECM), and biomaterials to fabricate 3D multicellular constructs in environments where cellular interactions (cell–cell, cell–ECM), biomechanical stresses (shear, pressure, stretch), bioelectrical signals, and soluble factor signaling (certain hormones, growth factors, cytokines) are all replicated to accurately model in vivo-like responses. “microphysiological systems” can be defined as “engineered multi-tissue/organ systems constructed using two or more tissue chips or by incorporating multiple interconnected “tissue”/”organ” chambers on one chip to recreate communications among different tissues, organs, and/or organ systems to model systemic interactions in the context of normal physiology, disease, or testing of candidate therapeutics.” Communication among “tissues” or “organs” incorporated in MPSs can be established through multiple approaches, depending upon the specific relationships of interest. For instance, aspects of the cardiovascular system can be recapitulated by employing flexible tubing (if connecting multiple TCs) or microfluidic channels (if all “tissues” or “organs” are housed on one chip), in combination with pumps, to provide for the perfusion of media among different chambers, thus allowing soluble factor signaling to take place as it would in vivo. Additional or alternative modes of communication can be enabled by incorporating aspects of the nervous, immune, endocrine, and lymphatic systems.

Both TCs and MPSs represent miniaturized versions of the human body and require microscale bioengineering technologies to organize cells into “tissues” and facilitate fluid flow. Therefore, microfabrication techniques and microfluidic technologies play an essential role in the construction and operation of TCs and MPSs. Each tissue type requires efficient design consideration to enable proper functionality. Design parameters include tissue architecture (cell types and organization, ECM composition), blood flow (oxygen and nutrient delivery, waste removal), and physical stresses (shear, pressure, stretch) associated with the target organ/tissue. This review focuses on providing a basic understanding of the tissue-level organization and function of four critical organs that are involved in metabolizing or excreting drugs (liver and kidney) or are frequently damaged by off-target drug toxicity (heart and blood vessels). Essential design considerations in the development of TC models of each organ/tissue are examined, and current efforts and past successes in TC models’ development are summarized. While this review focuses on the heart, vasculature, liver, and kidneys, more information on other organ systems can be found in the following excellent articles focused on the brain [6], lungs [7], GI system [8], pancreas [9], and reproductive system [10]. Additionally, the following review articles also provide comprehensive overviews of TCs and their use in drug testing [11,12] and disease modeling [13].

## 2. Cardiac Tissue Chips

### 2.1. Function

The heart is a complex organ responsible for pumping blood throughout the body and comprises four chambers: the right atrium, right ventricle, left atrium, and left ventricle. The atria are thin-walled cavities that receive blood from large veins (the vena cavae on the right and the pulmonary veins on the left) and act as reservoirs for the ventricles, the muscular chambers that abut the atria and contract after filling to propel blood forward [14]. The heart can be considered as two pumps that work in synchrony, with the right side of the heart involved in pumping deoxygenated blood from the systemic circulation into the lungs for oxygenation and release of carbon dioxide, and the left side of the heart receiving that oxygen-rich blood and pumping it forward to the rest of the body. Cardiomyocytes (CMs) contain actin, myosin, and other contractile proteins organized into sarcomeres, which contract and relax under the influence of ion gradients and membrane potentials [15]. While the primary function of the heart is to pump blood, atrial CMs also produce atrial natriuretic peptide (ANP) and ventricular CMs produce brain natriuretic peptide (BNP), hormones that both play roles in normal homeostasis of blood pressure and extracellular fluid volume [16].

### 2.2. Cell Types and Extracellular Matrix

Cardiac tissue is composed of many cell types, with the most abundant being CMs, fibroblasts (FBs), endothelial cells (ECs), and perivascular cells (which include pericytes and smooth muscle cells) [17,18,19]. The heart also contains two pacemakers and a conduction system composed of specialized CMs, as well as a robust intracardial nervous system [20]. The primary ECM proteins include different types of collagens, fibronectin, and elastin [21].

### 2.3. Cellular Organization

Cardiac TCs focus mainly on modeling the myocardium. In the native myocardium, CMs are organized in layers 2–5 cells thick and are surrounded by cardiac FBs within an ECM that contains mainly collagen. CMs assume a rod shape with predominantly end-to-end connections with neighboring CMs, called intercalated discs, and circumferentially distributed connections with the surrounding ECM [15,22]. Endothelial networks within the myocardium serve as conduits for blood to provide oxygen and nutrients and to remove carbon dioxide and waste.

### 2.4. Physical Stresses, Fluid Flow, and Electrical Signals

The heart constantly pumps blood and is, therefore, continually exposed to blood flow. However, the only layer that comes in direct contact with blood and experiences fluid shear is the endocardium. During the cardiac cycle, cells associated with all three layers of the heart (endocardium, myocardium, and epicardium) experience pressure and stretch. Pressure and stretch within a physiological range can promote physiological hypertrophy and enhance cardiac function. If applied in the pathological range, though, these stimuli frequently result in structural remodeling of the heart, which can ultimately lead to heart failure. Electrical signals generated in the sinoatrial (SA) node are propagated rapidly through the heart via the atrioventricular (AV) node and cardiac conduction system (CCS). Additionally, due to the presence of numerous gap junctions at intercalated discs, the myocardium functions as a syncytium, allowing for the rapid propagation of action potentials from depolarized CMs to adjacent resting CMs downstream, which leads to organized, nearly synchronous heart contractions [23].

### 2.5. Examples of Cardiac Tissue Chips

In vivo, CMs assume a rod-like morphology with predominantly end-to-end contacts to neighboring CMs (intercalated discs) and circumferentially distributed costameres which, in tandem with integrins, connect the CM contractile machinery to the surrounding ECM [15,22]. Several groups have used methods, such as microcontact printing [24] and nanotopographical surface cues [25] to direct the patterning of CMs in two-dimensional (2D) culture. Though 2D CM culture is relatively straightforward and amenable to high throughput applications, it is much less physiologically relevant than 3D culture. Native cardiac muscle is thick and composed of CMs interconnected in an intricate functional syncytium. Three-dimensional culture techniques can more faithfully replicate the cell–cell and cell–ECM contacts and associated mechanotransduction signaling seen in vivo than 2D CM culture models, which inherently lack many of these interactions [26,27,28]. Different spatial organizations of CMs in 3D can be accomplished via spheroid culture (without exogenous ECM) or seeding the cells in hydrogels, such as collagen I, fibrin, Gelatin Methacryloyl (GelMa), or alginate. CMs cultured as spheroids do not align longitudinally and frequently do not assume a rod-shaped morphology [28], while those cultured in hydrogels (primarily unidirectionally anchored hydrogels) self-assemble and align similarly to those in native cardiac tissue [29]. Cellular alignment and the formation of intercalated discs are facilitated by the uniaxial reorganization of the underlying ECM that occurs as the hydrogel compacts along lines of tension. While these 3D culture techniques capture more aspects of the in vivo CM niche, and can be adapted for co-culture with other cell types, the user has little control over how the cells organize themselves. Three-dimensional printing techniques provide new opportunities for defining cellular organization at high densities and for incorporating multiple cell types at pre-determined locations, allowing for higher control over cellular organization than methods relying on self-assembly.

**Figure 2:**
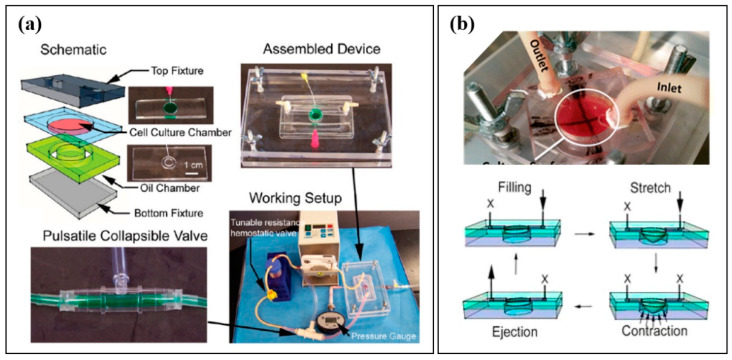
(**a**) Microfluidic Cardiac Cell Culture Model developed by Giridharan et al., along with a schematic diagram, images of an assembled device, pulsatile collapsible valve, and the complete working setup. Reproduced with permission from the American Chemical Society [32]. (**b**) Biomimetic Cardiac Tissue Model (BCTM), developed by Rogers et al., depicting the cardiac cell culture chamber and schematic diagrams representing how the BCTM reproduces the cardiac cycle. Arrows represent the direction of fluid flow and membrane stretch. Reproduced with permission from the American Chemical Society [34].

Native cardiac tissue undergoes a cycle of filling (diastole) and pumping (systole) during each heartbeat. The amount of blood ejected during each cycle (stroke volume) depends upon the degree of stretch induced in the left ventricle at the end of diastole (preload) and the load the heart must pump against to propel blood through the aortic valve (afterload). Increases in pressure and stretch often result in CM hypertrophy (increase in cell size) over time. Physiological hypertrophy is the increase in the size of the heart that occurs in the settings of development and exercise, whereas pathological hypertrophy (mainly affecting the left ventricular wall and, sometimes, the interventricular septum) occurs in the context of disease and is frequently associated with cardiac dysfunction [30]. Therefore, the culture of CMs under pressure and stretch regimens similar to those that occur during the cardiac cycle may be essential for reproducing some in vivo-like phenotypes and functions. As mentioned above, passive mechanical loads can be induced in 3D cultures by seeding cells in hydrogels, which are uniaxially anchored, usually between VELCRO^®^ fasteners (Velcro Companies, Manchester, NH, USA), other hook-and-loop adhesive strips, or posts of some variety. Over time, CMs will contract, compact the surrounding hydrogel, and generate increasing tension levels [29]. There are also several examples of efforts to deliver mechanical loads actively. In 2016, Marsano et al. introduced a microfluidic device containing an array of hanging posts to support fibrin gels. They used a pneumatic actuation system to induce uniform cyclic strain on 3D constructs consisting of neonatal CMs and hiPSC-derived CMs (hiPSC-CMs) [31]. In 2010, Giridharan et al., as a part of our Sethu research group, developed a microfluidic cardiac cell culture model (μCCCM) that mimicked the hemodynamic stresses incurred during pressure−volume changes in the left ventricle via the 2D culture of cardiac cells on a thin polydimethylsiloxane (PDMS) membrane situated at the bottom of a cell culture chamber (Figure 2a) [32]. The model enabled in vitro investigations of the effects of mechanical forces, similar to those seen during different stages of gestational development, on the functional maturation of CMs [33]. Within the μCCCM, a peristaltic pump filled the chamber with media, with the resultant downward pressure stretching the membrane and overlying cells (simulating diastole). An external pulsatile pneumatic pump generated an upward pressure, returning the stretched cell layer and membrane to their original position and transmitting much of this pressure into the culture chamber itself (simulating systole). In 2016, we modified this setup with an additional collagen I/Matrigel coating to adapt hiPSC-CMs to physiological hemodynamic stresses by gradually increasing the filling volume and “systolic” pressure over the course of 72 h [34]. In 2019, this biomimetic cardiac tissue model (BCTM) was further adapted in our lab to culture cardiac cells in 3D using fibrin gels suspended between two posts anchored on top of a PDMS membrane (Figure 2b) [35]. Using this setup, the researchers demonstrated that pressure–volume changes associated with cardiovascular development and disease could be accurately replicated in vitro.

Hansen et al. used a similar approach to generate engineered heart tissues (EHTs) of unpurified neonatal rat heart cells suspended in fibrin/Matrigel in rectangular casting molds in 2010 [36]. During gelation, a pair of PDMS posts suspended from above was lowered into the EHT, to which the construct then adhered while setting, allowing for its transfer out of the casting mold and into a medium-filled well. The researchers exposed the EHTs to known arrhythmogenic and cardiotoxic drugs, and the tissue strips demonstrated expected responses. For example, quinidine and erythromycin caused increases in relaxation time, and doxorubicin led to gradual decreases in contractile force. This EHT model has been validated for several applications with hiPSC-CMs and commercialized by EHT Technologies GmbH (University Medical Center Hamburg-Eppendorf, Hamburg, Germany) [37,38]. In 2016, Aung et al. developed a model capable of assessing CM contractile stress in real-time using soft lithography and microfluidic techniques [39]. In this TC, CMs labeled with a fluorescent marker were encapsulated in GelMa and seeded into a microfluidic device between two layers of non-degradable polyacrylamide gel filled with fluorescent tracking beads. The seeded device was then patterned via UV light exposure through a mask, eliminating CM-laden GelMa within microfluidic channels and other unwanted spaces. Deflected fluorescent signals from the bead-filled polyacrylamide gels served as “stress sensors” for the contractile stresses generated by the CMs’ contraction.

In addition to mechanical loading, electrical stimulation is another critical design consideration in cardiac TCs. In vivo, CMs contract in response to action potentials generated by SA node [40]. Initially, these impulses spread through atrial CMs, leading to a slow contractile wave, as well as to the AV node. The action potential is briefly stalled at the AV node (allowing the atria to complete contraction, thus emptying as much blood as possible into the ventricles before they contract), and is then propagated to the ventricular myocardium through the cardiac conduction system (CCS), which is composed of specialized CMs that conduct electrical signals much more quickly than normal CMs [41]. Thus, the ventricular CMs contract in near synchrony. Recently, progress has been made in differentiating SA nodal cells from hiPSCs [42] and generating pacemaker cells via the transfection of human mesenchymal cells with HCN1 [43]. However, the faithful recreation of the entire CCS will be challenging to achieve, and most cardiac TCs stimulate CM contraction via electrical pacing. The most common method for pacing CMs is field stimulation, where the stimulating current is applied uniformly to the tissue using a pair of carbon or platinum electrodes [44]. In 2018, Ronaldson-Bouchard et al. developed a platform incorporating field stimulation via carbon electrodes to induce the maturation of hiPSC-CMs in 3D culture [45]. They seeded hiPSC-CMs and FBs into a fibrin gel and cast the suspension into a mold between two flexible posts, which provided a passive mechanical load. By subjecting early-stage hiPSC-CMs (i.e., those which had just completed differentiation and begun spontaneously contracting) to progressively increasing frequencies of electrical stimulation, the researchers generated adult-like cardiac muscle over the course of a month.

To avoid the hassle of integrating bulky electrodes, some groups have developed optical stimulation techniques, which present simpler options for pacing CMs. CM contraction is ultimately dependent upon the influx and efflux of specific ions. Therefore, researchers have designed and incorporated light-sensitive ion channels (channelrhodopsins), allowing for pacing via optical illumination [46]. As an alternative, in 2018, Savchenko et al. developed graphene-based interfaces to optically stimulate CMs without the need for any modification to the cells [47]. This approach utilizes graphene sheets or flakes in proximity to CMs, which cause membrane depolarization in response to light stimulation. Additional methods allow for the direct electrical stimulation of CMs without the incorporation of bulky electrodes. For instance, Tandon et al. introduced surface-patterned electrodes to electrically stimulate CMs in 2010 [48]. They employed excimer laser ablation to microfabricate patterned microelectrode arrays (MEAs) using indium tin oxide (ITO), which is non-toxic, for the electrical stimulation of neonatal rat CMs and human adipose tissue-derived stem cells (hASCs). Their technique allowed for high-resolution patterning of virtually flat electrodes so that cells could be cultured on top of them in a monolayer. In 2012, Ma et al. incorporated an ITO-based MEA, along with a microfluidic device created via soft lithography, to investigate the electrical conduction between neonatal rat CMs and rat mesenchymal stem cells from bone marrow (rMSCs-bm), in comparison to conduction between CMs and control cells (CMs or FBs) [49]. After seeding CMs in the device channel and allowing them to mature into a 2D cardiac muscle fiber, the fiber’s central portion was scraped away with a glass pipet tip. rMSCs-bm or control cells were then seeded into the now barren central portion of the fiber via laser-patterning to allow single-cell resolution. Qian et al. also used a similar MEA TC design in 2017, together with an additional interdigitated electrode array, to fabricate a device to assess cellular electrophysiology, adhesion, and contractility in hiPSC-CMs [50]. In addition to the fine control over the electrical stimulation of cells, MEAs allow for measurements of extracellular field potentials. These measurements can be used to approximate intracellular action potentials, obviating the need for low throughput, user-intensive single-cell patch-clamps. Several lab groups have developed MEAs for recording action potentials from cells [50,51] and several companies (e.g., Multi Channel Systems MCS GmbH, Reutlingen, Germany; Maxwell Biosystems, Zurich, Switzerland; Axion Biosystems, Atlanta, GA, USA) offer commercially available MEAs that can be integrated into multi-well plates for quantitative cardiac electrophysiology analyses to screen drugs for potential cardiotoxicity. In 2014, Lin et al. developed a nanoelectrode array (NEA) that used nanoneedles to obtain intracellular patch-clamp recordings to increase these technologies’ overall throughput [52]. Furthermore, an increasingly popular technique to monitor cardiomyocyte electrical activity is the use of voltage-sensitive dyes (VSDs) or potentiometric dyes. Voltage changes within the culture alter the measurable spectral properties. This electrode-free approach is compatible with high throughput imaging platforms and has advantages in both 2D and 3D cultures [53].

While the nodes and CCS are ultimately responsible for creating and distributing the electrical impulses that cause the heart to contract, the cardiac autonomous nervous system (CANS) exerts significant influence over cardiac function [54]. Sympathetic and parasympathetic inputs to the SA node modulate heart rate. Meanwhile, inputs to the CMs affect parameters such as the force of contraction, the speed of relaxation, and the sensitivity to membrane polarization changes. The CANS has essential roles in normal physiology and is involved in numerous disease states, especially arrhythmias. Therefore, incorporating autonomic neurons into cardiac TC models increases their physiological relevance. In 2017, Sakai et al. developed a cardiac chip containing hiPSC-CMs cultured atop an MEA in one chamber and rat sympathetic neurons (rSNs) cultured in a separate chamber, with microtunnels connecting the two [55]. After 3–4 days in culture, axons extended through these microtunnels and made physical contact with the hiPSC-CMs. The researchers demonstrated that, with increased neuronal firing frequency (via direct stimulation by electrodes in the microtunnels), the hiPSC-CMs had a higher contraction frequency. This increase in contraction rate was dependent upon both the presence of sympathetic neurons and direct contact between these and the CMs. The addition of propranolol—a beta-adrenergic receptor blocker used clinically to slow the heart rate—to the CMs inhibited increases in beat frequency. This demonstrated that the neurons were influencing the CMs’ behavior in a physiologically relevant manner, increasing the chip’s applicability to disease modeling and screening proarrhythmic drugs. In 2016, Oh et al. co-cultured neonatal mouse ventricular myocytes (NMVMs) and sympathetic neurons differentiated from hiPSCs in the same culture chamber to improve hiPSC-derived neuron maturation and to confirm that they functioned appropriately and formed cell–cell contacts with the CMs [56]. Immunofluorescence confirmed the formation of neuron–CM connections, the importance of which was demonstrated in two ways. First, when the co-culture was supplemented with nicotine, the CMs appropriately beat more rapidly and with greater force. This result was not observed in control groups lacking the sympathetic neurons. Second, the researchers transduced the neurons with a channelrhodopsin-coding gene, allowing them to be controlled via photostimulation. When the neurons were stimulated via light to fire more frequently, the CMs responded with an increased contraction frequency unless propranolol was also present, in which case most of the rate increase was blocked.

As discussed previously, all CMs in vivo typically contract in response to electrical impulses generated in the SA node, and ventricular CMs rely upon the CCS to receive these action potentials. When the organized action potential generation and propagation is disrupted, CMs contract spontaneously as a result of their heightened electrochemical sensitivity and fluxes in intracellular calcium [57,58]. While adult human primary CMs and many CM cell lines do not spontaneously contract in vitro, CMs derived from hiPSCs, embryonic stem cells, and neonatal rodents retain this capacity [59]. Several research groups have sought to harness these CM populations’ ability to spontaneously contract, developing models that replicate the 3D ventricular structure and demonstrate the ability to pump fluid without the need for external pumps or electrical stimulation. Among the pioneers of this, Tanaka et al. developed a heart-on-a-chip pump that harnessed mechanical forces produced by spontaneously, synchronously contracting CMs to move fluid through a microfluidic channel in 2007 [60]. To achieve this, CMs were cultured as a monolayer until they demonstrated synchronous contractions generating significant force. The CM sheet was then detached and wrapped around a hollow elastomeric sphere, which contained inlet and outlet ports for the capillary channels and medium. In 2018, MacQueen et al. fabricated models of the left ventricle that more accurately mimicked ellipsoidal ventricular geometry via the pull-spinning of biocompatible, biodegradable polycaprolactone/gelatin nanofibers [61]. The ventricular scaffolds were seeded with either neonatal rat ventricular myocytes (NRVMs) or hiPSC-CMs, and both model types demonstrated synchronous chamber contractions 3–5 days after seeding. While the models had much lower cell densities than their in vivo counterparts and demonstrated relatively low ejection fractions and contractile work, they provided unrivaled geometric mimicry and synchronous contraction without the need for electrical stimulation. Additionally, when the researchers applied specific anatomical defects to the models, they observed stable pinned rotors and spiral waves, demonstrating their applicability for modeling arrhythmias secondary to structural defects.

### 2.6. Limitations

Currently available cardiac TCs do a reasonable job of mimicking cardiac tissue architecture with appropriate cell types and ECM, replicating electrical and mechanical loads, and modeling both normal and disease states. However, to mimic heart function and cardiac tissue behavior, it is essential that the engineered cardiac TCs pump fluid against an afterload. For this to occur, the contractile forces generated by the engineered tissue need to be sufficiently large. With current technology, the maximum forces generated are orders of magnitude smaller than what are seen in native cardiac tissue [62]. Finally, modeling cardiovascular disease using cardiac TCs can significantly benefit from integrating aspects of the immune system.

## 3. Vascular Tissue Chips

### 3.1. Function

The circulatory system transports blood throughout the body. There are two major circulatory networks in the body: the systemic and pulmonary vasculature. Within the systemic circulation, the arterial vasculature receives oxygenated blood from the left side of the heart and transports it to capillary networks throughout the body. Oxygen and nutrients are exchanged for carbon dioxide and waste products within the capillary networks, and the deoxygenated blood returns to the right side of the heart via the venous system. This deoxygenated blood is then transported via the pulmonary arteries to the lungs for oxygenation and the removal of carbon dioxide. This freshly oxygenated blood then returns to the left side of the heart via the pulmonary veins and is subsequently pumped back into the systemic circulation, completing the circuit. In addition to blood transport, the blood vessels play important roles in maintaining homeostasis within the body via the regulation of blood pressure [63], body temperature [64], and the facilitation of immune and endocrine functions [65].

### 3.2. Cell Types and Extracellular Matrix

Excluding the capillaries, blood vessels are composed of three tissue layers, the compositions and thicknesses of which vary depending upon the pressure and shear stress the vessel experiences. The inner layer (tunica intima) comprises a single layer of endothelial cells (ECs) and a thin layer of underlying connective tissue [66]. The intermediate layer (tunica media) consists primarily of smooth muscle cells (SMCs) and elastic fibers, and the outermost layer (tunica adventitia) is composed primarily of fibroblasts (FBs), along with lesser amounts of resident inflammatory cells, SMCs, and perivascular adipocytes. Capillaries consist of a single layer of ECs, an underlying basement membrane, and interspersed pericytes, and their function is to permit gas and nutrient exchange. The vascular ECM includes various proteins and macromolecules, with the most abundant being laminin, elastin, collagen, and fibronectin [67].

### 3.3. Cellular Organization

The ECs that form the tunica intima are roughly hexagonal, elongated along the blood flow axis, and cover the subendothelial collagen entirely, serving as an antithrombotic barrier [68]. In the tunica media, SMCs are organized in concentric layers with variable amounts of elastic fibers [69]. The smallest of the arterial vessels, the arterioles, have a thick tunica media and are heavily innervated by the autonomic nervous system [70]. The tunica adventitia is composed primarily of FBs and collagenous fibers and variable amounts of perivascular adipocytes [71]. The capillaries consist of only the endothelial layer, surrounded by a basement membrane.

### 3.4. Physical Stresses and Fluid Flow

Arteries are compliant blood vessels that experience higher blood pressures than other vasculature components due to their proximity to the heart. These vessels’ elastic nature leads to the dampening of the pulsatile flow of blood exiting the pumping heart, which provides a more uniform flow to downstream vessels. As arteries branch to distribute blood to various parts of the body, they become narrower, resulting in some diminution of blood pressure due to increased resistance. These vessels control the amount of blood flow to capillary beds and dramatically reduce blood pressure experienced by these delicate microvessels. Straight segments of arteries experience normal pulsatile flow. However, at locations near bends and bifurcations, adverse blood pressure gradients can develop, which lead to locally disturbed flow. Disturbed flow generates lower shear stress than normal laminar flow and can be classified as either oscillatory (bidirectional) or retrograde (reversed) [72]. These abnormal flow patterns are critical in the context of atherosclerosis, as plaques preferentially occur at locations that experience disturbed flow [73]. Accurately modeling arteries requires reproduction of physiologic (or pathophysiologic, depending on the model’s purpose) pulsatile pressure, stretch, and shear stress, along with local flow phenomena. Blood leaving the arterioles enters capillary beds containing multiple capillaries in parallel. The flow within these microvessels is significantly slower than in the arteries and veins, facilitating efficient gas and nutrient transfer. As blood exits the capillaries, it drains first into venules, which coalesce into larger veins that transport the blood back to the heart. Some exceptions to this general organization exist, such as the hepatic portal system, wherein venous blood passes through a second microvascular bed before coalescing once again into venules and veins to return blood to the heart. While veins and arteries experience similar flow velocities, the venule pressure is significantly less than in arterial vessels. This phenomenon is due to losses in pressure accrued during blood transit through high resistance arterioles and the venous circulation’s high compliance. As with the arterial vessels, modeling capillaries and venous vessels requires accurate replication of pressure, shear stress, and stretch to recreate physical stresses and fluid flow phenomena seen in vivo.

### 3.5. Examples of Vascular Tissue Chips

Some of the first vascular TCs were parallel-plate systems, which were initially developed in 1973, by Hochmuth et al. for investigating erythrocyte membrane elasticity [74]. This model was adapted in 1983 by C. L. Ives et al. and was used to examine the effects of different levels of laminar flow-generated shear stress on the orientation of arterial and venous ECs cultured in monolayers [75]. The parallel-plate flow chamber consisted of a glass slide or flat polystyrene surface, coated with various substrates, upon which the cells were cultured. A gasket helped secure the device, and an upper polycarbonate piece with inlet and outlet media ports was clamped to the other components, forming the flow chamber. This setup incorporated upper and lower media reservoirs on either side of the chamber. The hydrostatic pressure head due to the vertical distance between these fluid columns established and maintained laminar flow within the chamber. Flow rates could be altered by changing the vertical distance between the reservoirs or clamping the tubing upstream of the chamber, and a roller pump was employed to recirculate fluid from the lower media reservoir to the upper. Pulsatile flow could be introduced to this system by utilizing a cam-driven clamp flow oscillator, which pinches down on fluid-filled flexible tubing to propel the liquid therein. In 1985, Frangos et al. employed this adapted model to subject human umbilical vein ECs to a mean shear stress of 10 dyne/cm^2^ (within arterial physiologic range) to investigate the effects of steady versus pulsatile laminar flow on EC prostacyclin production and demonstrated that cells exposed to pulsatile flow generated 16 times the amount of prostacyclin [76]. When using parallel-plate flow chambers, the wall shear stress on the surface of the plate is calculated using the following equation:

(1) $$t_{wall}=\frac{\Delta Ph}{2L}=\frac{6Q\mu }{Wh^{2}}$$

where *Q* is the volumetric flow rate, *µ* is the absolute fluid viscosity, and *W*, *L*, and *h* are the width, length, and height of the rectangular flow channel, respectively [77]. Δ*P* is the pressure difference between the flow channel’s inlet and the outlet and can be manipulated by changing the media reservoirs’ vertical heights.

Non-uniform surfaces, such as ridges, steps, obstacles, or irregular patterns, can introduce disturbed flow [72,78]. One of the most frequently employed methods to create disturbed flow is to add a vertical step to the parallel-plate flow chamber by stacking two silicone gaskets with different size rectangular cutouts between the plates [79]. An uneven surface creates a drop-off between the media inlet and the EC monolayer, leading to predictable and reproducible vertically disturbed flow in the form of a recirculation eddy immediately following the step. Depending upon the step’s placement and height and the length of the EC monolayer downstream, one can investigate the effects of oscillatory disturbed flow, laminar flow, and the transitionary flow between all in one device [72]. Another standard method for generating disturbed flow utilizes a cone-and-plate viscometer with a rectangular obstacle placed on the stationary EC-covered plate. A cone-and-plate viscometer, used in industry to characterize the viscosity of fluids, utilizes a motorized cone with a very obtuse angle (on the order of 0.5°), the tip of which is oriented perpendicular to, and makes contact with, a stationary plate. To adapt this technology for vascular modeling, researchers have grown ECs in a monolayer on the plate and perfused media across the surface (via inlet and outlet ports placed on opposite edges of the plate). The cone spins and generates different flow profiles based on the cone’s angular velocity and any disruptions in the plate surface contour [80].

**Figure 3:**
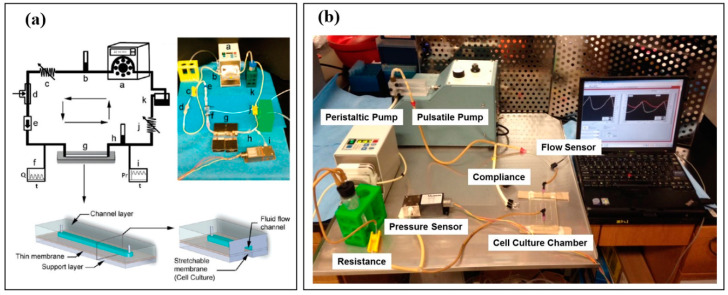
(**a**) Endothelial Cell Culture Model (ECCM), developed by Estrada et al., replicating physiological pressure, stretch, flow, and shear stress. The schematic diagram and system set up includes: a. peristaltic pump, b. pulmonary compliance, c. pulmonary resistance, d. collapsible chamber, e. one-way valve, f. inline flow sensor, g. cell culture chamber, h. aortic/systemic compliance, i. inline pressure sensor, j. aortic/systemic resistance, and k. medium reservoir. The schematic diagrams of the vascular chip within the ECCM show the cell culture chamber (bottom-left) and a cross-section view of the cell culture chamber showing the thin membrane on which cells are cultured (bottom-right). Reproduced with permission from the American Chemical Society [82]. (**b**) Modified ECCM setup replicating both normal pulsatile flow and continuous flow, as seen in CVAD usage. Reproduced with permission from [83,84].

Beyond modeling different flow profiles, replicating the blood pressure and stretch that ECs experience in vivo is also essential. The previously mentioned models do not account for EC strain, as the plates the cells reside upon are rigid and flat to ensure the precise tuning of flow patterns. In 2011, Estrada and others from our Sethu lab group sought a more holistic approach to vascular TCs with the Endothelial Cell Culture Model (ECCM) (Figure 3a), which can simultaneously replicate normal or abnormal flow profiles, pressure, shear stress, and stretch in a closed flow loop setup [81,82]. The model consisted of a predesigned EC culture chamber, a pulsatile flow pump to generate adjustable pulsatile flow, a peristaltic roller pump capable of maintaining continuous fluid flow, a one-way flow control valve to prevent retrograde flow, a compliance element to modulate pulse pressure, a tunable flow resistance element to mimic systemic arterial resistance, and a fluid reservoir, along with pressure and flow sensors. The EC culture chamber was a microfluidic device fabricated using a 3D-printed mold containing the chamber channels’ negative impressions. To create the device, PDMS was poured into the mold and cured to yield the upper piece of the chamber, which formed each channel’s top and sides. After punching an inlet and outlet port for cell seeding and media access, the top piece is then bonded onto a thin, flexible PDMS membrane via oxygen plasma treatment to create a channel. Disturbed flow, similar to the infrarenal segment of an atherosclerotic abdominal aorta, was generated in this model by removing the one-way valve (thus allowing retrograde flow) and lowering the flow rate to mimic the flow velocity and shear stress experienced by the aortic ECs in vivo.

In addition to modeling disturbed flow profiles, our lab group has used the ECCM in two studies to compare the effects of arterial physiologic pulsatile fluid flow on arterial ECs to those of continuous flow, as seen in patients with a continuous-flow ventricular assist device (CF-VAD) (Figure 3b) [83,84]. We replicated physiologic arterial flow in vitro by tuning the pump settings and resistance and bypassing the compliance element, generating peak systolic/diastolic pressures of approximately 120/80 mmHg, 6–8% stretch, and a 9 mL/min flow rate at a pump frequency of 80 beats/min. By incorporating the compliance element, pulse pressure was reduced to about 7 mmHg (peak systolic/diastolic pressures of 99/92 mmHg), while stretch and mean flow rate matched that of the pulsatile model. In 2019, Haglund and others from our group also demonstrated that synchronous and asynchronous flow profiles similar to those of popular CF-VAD models could be replicated, with the synchronous flow requiring some alteration of the compliance element, and the asynchronous flow necessitating the addition of a second pulsatile pump [84]. In a recently accepted but not yet published study of ours, physiologic arterial flow parameters of 120/80 mmHg pressure, 6–10% EC strain, 15 dynes/cm^2^ average shear stress, and 10–12 mL/min flow rate at 60–80 beats/min was achieved [85]. They also demonstrated CF-VAD-like parameters of 100 mmHg pressure (pulse pressure completely eliminated) and 12 mL/min flow rate within the same system. The ECCM, and other models that can replicate multiple aspects of normal and abnormal circulation, are promising and powerful tools, as they allow for direct comparisons between physiologic and pathophysiologic settings. These TCs can help the scientific community better understand disease progression due to circulation abnormalities and serve as platforms for testing therapeutics aimed at vascular complications.

While the above 2D models have been useful in elucidating the effects of specific circulation parameters on ECs at the cellular and molecular levels, they cannot fully replicate many aspects of native vasculature, including the 3D cell–cell and cell–ECM interactions that affect cell differentiation and survival, limiting their accuracy and applicability in studies of angiogenesis or cytotoxicity [26,86,87]. Steven George and his colleagues have made substantial progress over the past several years in creating 3D microvascular TCs that mimic in vivo capillary beds in form and function due to these models’ perfusable nature. These vascular TCs have proven to be instrumental platforms for investigating normal and pathologic angiogenesis in complex 3D microenvironments. In the first published iteration of this model in 2013, Moya et al. employed soft lithography and photolithography to create a PDMS microfluidic device consisting of a centrally-located row of 12 interconnected microtissue chambers [88]. The channels communicated via pores on opposite chamber edges to mimic the in vivo organization of capillary beds between arterioles and venules. Human endothelial colony forming cell-derived ECs (ECFC-ECs) and normal human lung FBs (NHLFBs) were seeded into the device in fibrin, a pro-angiogenic protein found in the provisional matrix of granulation tissue during the wound-healing process. The pores connecting the tissue chambers to the microfluidic channels were curved to prevent fibrin from escaping into the channels before gelling. Following seeding and fibrin gelling, flow along the channels and interstitial flow across and between the tissue chambers were established by connecting media reservoirs with differing fluid column heights to the media inlet and outlet ports. The height difference created a hydrostatic pressure head along each channel and across each tissue chamber between the high pressure “arteriole” side and low pressure “venule” side. The investigators also incorporated a removable “jumper” tube, which enabled the flow of media directly from the “arteriolar” channel to the “venular” channel. Using this model, one can control fluid flow rates along the channels, between the channels (across the tissue chambers), and among the tissue chambers by merely altering the media column heights and utilizing the jumper tube, enabling the tuning of the shear stress and solute gradients experienced by the cells within. With this TC, the researchers established self-assembled, robust capillary beds within 2–3 weeks, which were anastomosed to the microfluidic channels (allowing perfusion through the vessel lumen) by periodically reversing the pressure gradients between the channels. NHLFBs were incorporated in this model because, in vivo, FBs work in concert with ECs to generate the endothelial basement membrane. These cells secrete pro-angiogenic growth factors, principally vascular endothelial growth factor (VEGF) and basic fibroblast growth factor (bFGF or FGF-2). As VEGF and bFGF were omitted from the media during the first 14 days after device seeding, the successful self-assembly of microvessels in this model was attributable to interstitial flow gradients, which are known to influence angiogenesis in vivo due to effects on cell migration, ECM remodeling, and vascular sprout formation, and the presence of growth factor-secreting stromal cells. After the capillary beds were fully formed and perfusable, VEGF and bFGF were added to the media to make them more robust. Perfusion through the capillary networks, both between the “arteriole” and “venule” channels across the capillaries and among the interconnected capillary networks, was evaluated and confirmed by adding 1 μm fluorescent beads to the media during the third week of culture. While the permeability of the vessels was investigated using fluorescent dextran of known molecular weights [89,90]. These 3D microvascular TCs are lower throughput than many of the previously described 2D models, diminishing their usefulness in applications such as drug screening. However, they have proven useful in expanding our understanding of physiologic and pathophysiologic angiogenesis. Lastly, they have provided a jumping-off point for the vascularization of engineered tissues, which can help overcome the oxygen diffusion limitation that currently hinders the ability to generate constructs thicker than a few hundred μm [91,92].

Due to the low throughput of these self-assembling TCs and other similar designs, some researchers have sought to create 3D perfusable vessels by lining microfluidic channels with ECs. While native blood vessels have a circular or ovoid cross-section, most vascular TCs have a rectangular cross-section for several reasons. First, flat surfaces are easily reproducible, and tissue engineers can readily culture cells on a flat substrate. Secondly, most vascular TCs are fabricated with PDMS, due to its transparency, tunable elasticity, and biocompatibility, via a combination of soft lithography and photolithography, with the latter being employed in device mold construction. One technical limitation of photolithography is that it does not allow for the fabrication of easily reproducible rounded corners. Additionally, even if the user were to achieve a somewhat circular channel mold via photolithography or 3D printing, the resultant PDMS device must be bonded to another surface to complete the channel. Another downside to models wherein the vascular channels are completely surrounded by PDMS is that vessels, in vivo, are surrounded by ECM and other cells. To address these limitations, some have employed bioprinting to create endothelial channels of defined architecture surrounded by ECM. For example, in 2016, Zhang et al. used sacrificial bioprinting to create a vascular TC with microvascular channels lined by ECs and FBs, embedded in GelMA ECM [93]. To model thrombosis, researchers bioprinted a bifurcated vessel (thromboses frequently occur at such sites in vivo) and infused the model with human blood. They then induced clotting in one branch of the bifurcation and analyzed the flow through the open stem vessel and patent bifurcation branch, which showed similar flow velocity reductions to those seen in vivo. In 2018, Schöneberg et al. also employed bioprinting to generate perfusable vessels composed of ECs, fibroblasts, and smooth muscle cells, with diameters of 1 mm [94]. These bioprinted vessels more closely mimic small arteries, and over 80% of cell viability was demonstrated after 3 weeks.

### 3.6. Limitations

Current vascular tissue chips are capable of recreating planar or tubular vessel models with appropriate vascular cell types. Several flow setups have also been developed to recreate blood flow phenomena and vascular bed-specific flow conditions. However, a majority of these flow setups utilize planar monolayer cultures supported using artificial materials and scaffolds. More accurate replication of in vivo phenomena requires developing 3D tubular structures that can be integrated within these flow setups and are capable of sustaining in vivo levels of pressure and stretch for physiologically relevant disease modeling.

## 4. Liver Tissue Chips

### 4.1. Function

The human liver is the largest internal organ of the body and carries out various functions. It plays a pivotal role in the maintenance of glucose levels; metabolizes drugs, lipids, and amino acids; it functions as an excretory organ for bile and cholesterol; enables endocrine function by altering hormone and degrading circulatory hormones; lastly, the liver stores glycogen, fat-soluble vitamins, and iron [95].

### 4.2. Cell Types and Extracellular Matrix

The liver parenchyma is composed of specialized epithelial cells called hepatocytes (HCs), and the stroma consists of hepatic stellate cells (HSCs), which are specialized mesenchymal cells, resident macrophages called Kupffer cells (KCs), fenestrated liver sinusoid endothelial cells (LSECs), and a small number of cholangiocytes, which line the bile ducts. These resident cells are all necessary for maintaining the organ’s complex signaling pathways and metabolic environment, allowing the liver to perform integral functions regarding synthesis, filtration, detoxification, and metabolism [96]. The liver’s ECM consists mainly of collagens I, III–VI, as well as fibronectin and laminins [97].

### 4.3. Cellular Organization

The hepatic lobule is the structural and functional unit of the liver. Each polyhedral lobule consists of hundreds of irregular plates of HCs (“hepatic cords”) radially projecting outward from a terminal hepatic venule (“central vein”) toward about six peripherally located portal tracts, each of which contains a hepatic arteriole and portal venule, as well as a bile ductule [98]. Due to the morphologically homogenous nature of liver parenchyma and the fact that somewhat irregularly spaced portal tracts delineate lobules, definitive measurements of lobule size have proven challenging to acquire, but each is believed to have a volume of ≤1 mm^3^ [99]. Blood flows from the portal tract to the central vein via sinusoids, which are low-pressure, fenestrated vascular channels lined by LSECs with minimal or no basement membrane. The sinusoids receive oxygen-rich, nutrient-poor blood from hepatic arterioles and nutrient-rich, oxygen-poor blood from portal venules. As blood flows through these permeable vascular channels toward the central vein, plasma flows into and out of the perisinusoidal space, providing oxygen and nutrients to the adjacent hepatic cords and allowing hepatocytic secretory products and waste to enter the sinusoids, and eventually, the systemic circulation [100]. KCs reside in the lumen of the sinusoids, scavenging microbes, particulate debris, and dying erythrocytes from the blood, and HSCs reside in the perisinusoidal space, storing fat, vitamin A, and regulating sinusoidal tone. Though HSCs, under normal conditions, are mostly senescent, maintaining ECM homeostasis, they respond to insult via replication and increased collagen production, leading to fibrosis.

While blood flows from the periphery of the lobules to the central veins, bile flows in the opposite direction through canaliculi, formed by the adherent apical membranes of HCs [101]. HCs secrete bile into the canaliculi, where it travels to the bile ductules in the portal tract, which are lined by cholangiocytes that modify the substance through absorption and secretion. These ductules coalesce into larger diameter ducts, and the bile eventually exits the liver via the common hepatic duct.

### 4.4. Physical Stresses and Fluid Flow

Blood flow through the liver from the portal venules and hepatic arterioles of the portal tracts into the hepatic sinusoids results in fluid shear stress on the LSECs [102]. Increased shear stress within the sinusoids can negatively impact the LSECs, leading to the enlargement of fenestrae through fusion and resultant disturbed microcirculation between the sinusoidal and perisinusoidal space. HCs secrete bile, which is transported via canaliculi, which are about 1.4 μm wide and eventually drain into the portal tracts’ bile ductules, presumably resulting in very low shear stress [103]. Various disease states can increase the viscosity of bile, resulting in elevated shear stress.

### 4.5. Examples of Liver Tissue Chips

There are several challenges associated with the culture of HCs in vitro, as they quickly dedifferentiate under standard monolayer culture conditions, leading to a loss of polarity. Since HCs secrete biliary components into bile canaliculi via their apical domains while secreting and absorbing different molecules via their perisinusoidal basal domains, it is not surprising that this loss of polarity leads to dramatic decreases in hepatocyte-specific functions, such as albumin secretion, ammonia metabolism, and the expression of drug-detoxifying enzymes [104]. Modeling the liver microenvironment using TCs can help preserve the HC phenotype, averting the dedifferentiation-associated challenges seen in standard cell culture systems. For example, HCs cultured under physiologic hepatic flow conditions demonstrate more vital nutrient and waste exchange than those cultured in static conditions. Additionally, HCs in vivo display specific phenotypic hallmarks, based on proximity to the central vein versus the peripheral portal tracts. These characteristics can be replicated in vitro if the cells are cultured under hormone and oxygen gradients similar to those experienced in the lobules [96]. For the purposes of this review, liver TCs are classified into one of the following categories: 2D planar, 3D spheroid culture with/without matrices, hanging drops, 3D-printed, layer-by-layer deposition, and microwell systems.

Liver TCs based on 2D planar culture are high throughput due to the relative ease of culturing cells on flat surfaces, but most of these models fail to recreate HC polarization and the complex signaling milieu among HCs and the various resident stromal cells. Co-culturing HCs with one or more stromal cell types can replicate in vivo cell–cell interactions to some degree when micropatterning technologies are employed. However, the success of this patterning and the preservation of differentiated HC phenotypes are highly dependent on the cell culture substrate, choice of stromal cells, and the degree of homotypic (between like cells) and heterotypic (between distinct cell types) interactions [105,106]. In 2013, Ho et al. recreated the 2D morphology of hepatic lobules (namely, the radial organization of flat HC cords with lines of ECs interspersed in between, with acellular areas left between to mimic the perisinusoidal space) by micropatterning co-cultured HCs (HepG2 cell line) and human umbilical vein ECs [105]. Their culture substrate was a glass wafer coated with collagen I to aid cell attachment and patterned with two distinct electrodes for cords of HCs and ECs. After adding silicone tape to the edges to act as a spacer, ITO glass was added atop the device to act as a ground electrode. The cells were then sequentially seeded onto the micropatterned wafer, with enhanced field-induced dielectrophoresis (DEP) used after each respective seeding step to guide and trap the cells on the appropriate patterned electrodes. The researchers observed successful patterning, 95% viability, and 80% enhancement of CYP450-1A1 enzyme activity (integral in hepatic drug metabolism processes) compared to non-patterned HepG2 culture after two days. While faithful recreation of lobular architecture may require complex patterning procedures, more straightforward patterning methodologies can still lead to impressive results, as demonstrated by Khetani and Bhatia’s 2D micropatterned co-culture model (MPCC), which they developed in 2008 [106]. MPCC fabrication utilized photolithography (or outsourced mold fabrication), soft lithography, and oxygen plasma ablation to create circular “islands” of type I collagen, uniform size, and spacing. Primary HCs, which require ECM for cell culture adherence, are seeded first and attached to these islands. After allowing these cells to attach and proliferate for several hours, the cell culture substrate is washed (to prevent potential non-specific HC adherence). The 3T3-J2 murine FB (immortalized cells that have been shown to optimally preserve primary HC phenotype) are then seeded and proliferate to fill the non-collagen-coated areas. Using this model, researchers have demonstrated the maintenance of primary HC phenotypes, including albumin and urea production, physiologic CYP450 enzyme subtype activity and expression levels, and the presence of bile canaliculi, over the course of several weeks. Though primary rat HCs were used initially, the MPCC has been refined for human HCs, and has been used for drug screening applications, as well as for the investigation of disease mechanisms involving hepatitis B, hepatitis C, and *P. falciparum* [107,108].

**Figure 4:**
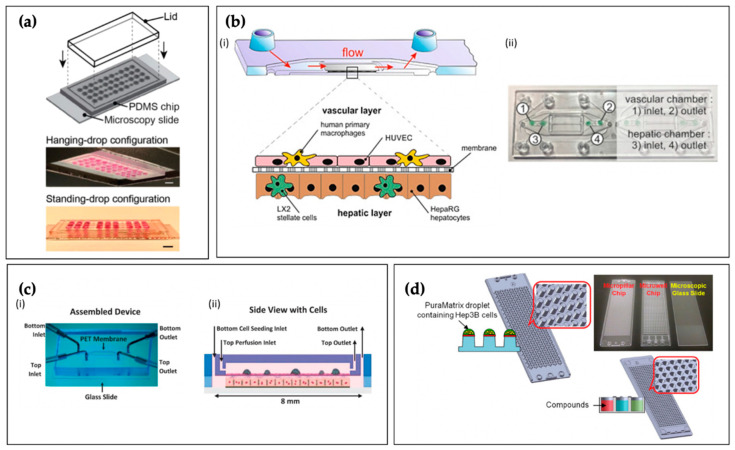
(**a**) Boos et al.’s microfluidic liver hanging-drop platform patterned on the surface of a PDMS substrate, bonded to a microscopy slide, and a lid is inserted into a groove structure to cover the open system. Scale bar: 5 mm. Reproduced with permission from [111]. This work was published under a CC BY license (Creative Commons Attribution 4.0 International License; https://creativecommons.org/licenses/by/4.0/); (**b**) Design of microfluidically-perfused liver biochip with a vascular layer composed of ECs and tissue macrophages, and a hepatic layer comprising HSCs co-cultured with HCs (**i**). Biochip device denoting inlet and outlet ports (**ii**). Reproduced with permission from Elsevier [112]; (**c**) Assembled two-chambered microfluidic device separated by a porous membrane (**i**), and a side-view schematic of the device with the four cell types: primary human HCs, EA.hy926 (human umbilical vein ECs), LX-2 (human HSCs), and U937 cells (human macrophage cell line) (**ii**). Reproduced with permission from John Wiley and Sons [113]; (**d**) Schematic representation of the micropillar and microwell chip platform with 3D-cultured Hep3B cells encapsulated in PuraMatrix™ for compound hepatotoxicity assessment. Reproduced with permission from Elsevier [114].

Though recent advancements in 2D hepatic models have improved their usefulness, they fail to capture many important aspects of the native 3D liver architecture. Three-dimensioanl hepatic spheroid/microtissue cultures show enhanced metabolic and toxicological phenotypes, leading to superior sensitivity and specificity in detecting known hepatotoxins, compared to 2D sandwich cultures [109]. Scaffold-free (i.e., matrixless) 3D cell spheroids can be generated via multiple routes, such as the hanging drop method, which utilizes gravity and the inherent strength of cell–cell attachments to achieve uniformly sized cell aggregates. Hepatic spheroids can also be generated by culturing cells on specialized substrates, such as cell-repellent plates. Additionally, by employing magnetic beads in cell culture and applying mild magnetic forces, spheroids of uniform size can be rapidly produced, and these cell aggregates can be conveniently and reversibly immobilized for various downstream assays [110]. In 2019, Boos et al. employed soft lithography to fabricate a microfluidic device enabling the “co-culture” of primary human HC spheroids and murine embryoid bodies, both generated via hanging drop technique (Figure 4a) [111]. Though these distinct cell aggregates were cultured in separate chambers, the wells were connected via microfluidic channels. Gravity-driven flow (induced by periodic tilting of the TC) generated continuous communication between the tissues, constant medium turnover, and immediate exchange of metabolites. The purpose of this study was to improve the reliability of embryonic stem cell testing, an animal-free method of screening drugs for teratogenicity, by incorporating physiologic hepatic drug metabolism. Though the HCs in the spheroids maintained their phenotype for over 10 days, an appropriate duration for this specific drug screening application, the longer-term culture of hanging drop spheroids is limited, hampering their utility in chronic toxicity or liver disease studies.

Many 3D liver TC models utilize cells embedded in a natural or synthetic matrix due to the lack of control over cellular organization in spheroid culture. Suspending cells in liquid ECM before gelation also facilitates seeding into specific locations in microfluidic devices, which is difficult to achieve when working with spheroids. For example, Toh et al. in 2009 fabricated a 3D chip containing primary rat HCs (3D HepaTox Chip), which incorporated eight separate cell culture chambers and a microfluidic concentration gradient generator [115]. This gradient allowed the culture chambers to be perfused with media containing eight varying drug concentrations. Each culture chamber had a central compartment for cells, surrounded by a micropillar array, and bordered on each side by a medium perfusion channel. To seed the HCs into the culture chambers, liquid HC/methylated collagen suspension was added into a common reservoir (which was connected to the eight different cell culture chambers). Negative pressure was then applied to an outlet port on the opposite side of the device (also connected to the cell culture chambers), and gelation was achieved by infusing anionic HEMA-MMA-MAA terpolymer into the perfusion channels bordering each cell culture chamber. With this system, the researchers exposed primary rat HCs to five different drugs (acetaminophen, diclofenac, quinidine, rifampin, and ketoconazole) at different concentrations for 24 h and then assessed hepatotoxicity based on cell viability. The researchers also examined HC function (albumin production, phase I/II drug metabolic activity) with or without drug exposure. Compared to their multi-well plate 2D controls, HCs in the 3D HepaTox Chip demonstrated comparable, if not elevated, phase I/II metabolic activity and albumin production over 72 h. They also found that the IC_50_ value (the drug concentration that inhibits 50% of cellular activity) of each drug tested was comparable to previously reported values for primary rat HCs. Additionally, the *R*^2^ correlation between the IC_50_ values they observed and reported LD_50_ values (the drug concentration that kills 50% of exposed subjects) was more robust than the correlation seen when using HC cell lines instead of primary cells. While seeding cells in ECM can enhance cell–cell and cell–matrix interactions and preserve HC viability and phenotype for prolonged periods of time, care must be taken to choose the appropriate matrix and to minimize issues such as potential immunogenicity, batch-to-batch variation, and perturbed signaling pathways (due to release/absorption of soluble factors) [96].

Some of the previously described liver TC models have limited throughput and are not feasible for many labs due to the complexity of their fabrication methods and seeding protocols. They also frequently sacrifice the faithful recreation of hepatic architecture due to the difficulty of patterning multiple cell types on a microscale, especially in three dimensions. Though it does not address the accessibility issue, 3D bioprinting allows for more accurate replication of hepatic lobule architecture by utilizing programmable printing paths for multiple cell types, and the automated nature of this technology increases throughput and the reproducibility of results. Combining 3D-bioprinting of multiple hepatic cell types with microfluidic perfusion is a promising strategy for maximizing the physiologic relevance of liver TCs [96]. In 2016, Norona et al. utilized a 3D-bioprinting approach to create human liver constructs consisting of primary HCs, HSCs, and human umbilical vein ECs, and then repeatedly exposed these engineered tissues to low dosages of the hepatotoxic, fibrosis-inducing drugs methotrexate and thioacetamide [116]. As liver fibrosis generally develops over a prolonged time course, realistic in vitro modeling necessitates long-term viability. An additional hindrance to modeling liver fibrosis is that HSCs, the primary mediators of this condition, tend to become activated in vitro, generating a fibrotic response even without exposure to hepatotoxins. Both of these issues were addressed by this model, as the constructs in this study survived for four weeks, and HSCs maintained a quiescent phenotype prior to drug exposure. These 3D-bioprinted tri-cultures demonstrated appropriate and distinct pathological responses to the fibrosis-inducing agents, enabling the more comprehensive characterization of drug-induced liver injury mechanisms and improved compound risk assessment. Similar to other cell printing technologies, liver-bioprinting is sensitive to the printer’s accuracy, is expensive compared to other fabrication strategies, and the user may encounter challenges in achieving single-cell resolution precision, although next-generation devices offer this capability [117,118,119].

While 3D bioprinting allows for unrivaled architectural precision, channels within the liver (perisinusoidal space, zonal regions, and vasculature) can be mimicked via less expensive 3D culture methods, such as the layer-by-layer approach. For example, Rennert et al. in 2015 integrated HCs, HSCs, human umbilical vein ECs, and macrophages into a perfusable microfluidic TC to enable sufficient nutrition supply and to recapitulate the liver’s microenvironment (Figure 4b) [112]. The model incorporated a microporous membrane as a cell culture substrate, and the researchers established a co-culture of macrophages and ECs on one side (mimicking the KCs and LSECs of the hepatic sinusoids) and HCs and HSCs on the other, with the membrane acting as the perisinusoidal space. This arrangement elicited the hepatic lobule’s morphological aspects, such as the formation of HC microvilli extending toward the EC/macrophage “sinusoid” and increased hepatobiliary secretion. In 2016, Prodanov et al., using a similar approach, developed a TC with two microfluidic chambers, separated by a porous membrane, mimicking the sinusoidal microarchitecture, which remained viable over 28 days and demonstrated high albumin synthesis and urea excretion compared to static controls (Figure 4c) [113].

Lastly, microwell systems, which consist of numerous wells with μL scale volumes, allow for high throughput and the large-scale screening of hepatoxic compounds by immobilizing small numbers of cells on micropillars and traps. In 2018, Roth et al. tested the ability of seven different polymer coatings to aid in the attachment of PuraMatrix™ peptide hydrogel (Corning Inc., Corning, NY, USA), to micropillars in a microwell array. They then 3D cultured Hep3B human HCs (60 nL volume, 360 cells per pillar) for the high-throughput assessment of compound-induced hepatotoxicity and adenoviral transduction efficiency (Figure 4d) [114]. The researchers established a high-throughput liver TC for rapid toxicity assessment that was receptive to adenoviral transduction through the successful attachment of the nanoscale PuraMatrix™ embedded HC aggregates to the micropillar array.

### 4.6. Limitations

Current efforts to develop liver TCs have been successful in replicating a subset of liver-specific functions. However, the liver is a complex organ that performs numerous functions and relies on input from multiple organs and systems in the body. Physiologically relevant liver TCs need to closely mimic the native liver architecture as well as inputs and interactions with other organs and systems in the body to accurately replicate all aspects of the liver’s function. As the liver also plays a key role in modulating immune responses, the integration of the immune system components may be necessary to accurately model liver-related functions.

## 5. Kidney Tissue Chips

### 5.1. Function

The kidney’s primary function is to remove waste products and excess fluid from the body, actively establishing and maintaining homeostasis. As blood from the afferent arteriole flows through the glomerular capillaries, Starling forces (hydrostatic pressure and oncotic pressure gradients) cause much of the plasma to pass into Bowman’s space as glomerular filtrate [120]. Together, the capillary endothelium, basement membrane, and foot processes of the podocytes form the filtration barrier [121]. From here, the filtrate travels down the proximal tubule where a majority of water, salts, and organic solutes are reabsorbed into the peritubular capillaries. The filtrate then enters the loop of Henle where more water and Na^+^ and Cl^−^ ions are reabsorbed, followed by the distal convoluted tubule. Although not technically part of the nephron, the collecting duct system follows the distal tubule, and it is the site where Ca^2+^, Na^+^, and Cl^−^ are actively reabsorbed. The remaining filtrate is excreted as urine through the renal calyces.

### 5.2. Cell Types and Extracellular Matrix

The nephron is the kidney’s functional unit, and each kidney contains approximately 1.2 million of them [121]. Each nephron consists of a plasma filtration unit called the renal corpuscle, which is composed of a specialized tuft of capillaries (the glomerulus) and the surrounding Bowman’s capsule, and a segmented renal tubule, which includes a proximal tubule, the loop of Henle, a distal tubule, and a connecting tubule, which connects to the collecting tubules and ducts. The renal corpuscle consists of four different cell types, including the glomerular endothelial cells (GECs) and mesangial cells (MCs) found within the glomerulus, the podocytes of the visceral Bowman’s capsule layer, and the parietal epithelial cells (PEpCs) of the parietal Bowman’s capsule layer. The PEpCs are in continuity with the renal proximal tubular epithelial cells (RPTEpCs). The glomerular ECM primarily consists of collagen type IV, laminin, and heparan sulfate proteoglycans. The tubular ECM is predominantly made up of different types of collagens, glycosaminoglycans, polysaccharides, and glycoproteins, such as fibronectin.

### 5.3. Cellular Organization

The renal corpuscle is essentially a plasma filtration barrier and consists of three layers, the fenestrated capillary ECs, the glomerular basement membrane (synthesized by the capillary ECs and podocytes), and the interdigitated pedicles of the podocytes. The renal tubule consists of sequential tubular segments composed of tubular epithelial cells (TEpCs) of varying morphologies. The proximal tubule is lined by cuboidal epithelium, with an extensive brush border of microvilli. The loop of Henle contains a flat squamous epithelium, and the distal convoluted tubule and connecting tubule consist of cuboidal epithelium. The basal aspects of the TEpCs are anchored to a basement membrane. All epithelial cells within the nephron and collecting duct, except for the intercalated cells, have an apical single and nonmotile primary cilium that extends into the fluid [121]. These primary cilia work as mechanosensors and chemosensors, sensing changes in flow rate and chemical compound alterations, respectively. Though not strictly considered a portion of the nephron, the peritubular capillaries and vasa recta lie alongside the renal tubule and actively participate in secretion and reabsorption [120].

### 5.4. Physical Stresses and Fluid Flow

The biomechanical forces within the nephron, including the pressure, circumferential stretch, and fluid shear stress, are integral to several distinct signaling pathways and transport phenomena. Furthermore, tubular flow rates are dynamic and vary dramatically between different nephron segments [122]. Alternations in flow rate occur in concert with changes in glomerular filtration rate (GFR), tubuloglomerular feedback (TGF) via the macula densa, pelvic wall contraction, and reabsorption rates [123,124,125,126,127,128]. In general, TEpCs experience a fluid shear stress an order of magnitude smaller than endothelial cells (less than 1.0 dyne/cm^2^) [123,129]. The average tubular pressure decreases along the nephron, from approximately 13 mmHg in the proximal tubule to  less than 7 mmHg in the collecting duct [129,130]. Within the glomerulus, ultrafiltration from the glomerular capillaries into Bowman’s space occurs due to Starling forces. The approximate net ultrafiltration pressures are 17 mmHg on the afferent end and 8 mmHg on the efferent end [121]. In response to vasoconstrictors and vasodilators, the nephron will alter shear stress and stretch to ultimately influence the GFR and the renal blood flow [131,132].

### 5.5. Examples of Kidney Tissue Chips

One of the primary goals of kidney TC engineering is developing platforms for high-throughput, the reliable assessment of drug toxicity affecting the renal proximal tubule. RPTEpCs are very metabolically active, thus requiring a near-constant energy supply, and are continuously exposed to high concentrations of drugs and their toxic metabolites, making them highly susceptible to damage. The first human kidney TC model developed by Jang et al., in 2013, consisted of a porous polyester membrane coated with collagen IV sandwiched between two PDMS slabs [133]. The upper PDMS slab contained inlet and outlet ports for media and cells and a flow channel directly atop, and in contact with, the collagen-coated porous membrane. The lower PDMS slab contained a media reservoir, with ports for sampling and adding media/compounds, directly below and in contact with the porous membrane’s unmodified basal surface. After seeding the apical (upper) side of the membrane with RPTEpCs and allowing them to grow into a confluent monolayer, the cells were exposed to physiologic shear stress by flowing media through the upper PDMS channel. This device configuration mimics the in vivo architecture of the proximal tubule. The upper PDMS flow channel and underlying cell-covered porous membrane represent the tubule’s lumen and wall, respectively, with the media reservoir in the lower slab acting as the interstitial space surrounding the tubule. Compared to static controls cultured in Transwell^®^ (Corning Inc., Corning, NY, USA) systems, RPTEpCs subjected to physiologic flow showed enhanced epithelial polarization and primary cilia formation, indicating that cells were recapitulating in vivo organizational characteristics. RPTEpCs that experienced flow demonstrated more significant albumin transport, glucose reabsorption, and brush border alkaline phosphatase activity. Furthermore, RPTEpCs exposed to cisplatin (via the “interstitial” lower media reservoir) in the flow model more closely mimicked in vivo responses to the drug than those in static culture. Though this model represented a significant advancement over traditional culture systems, it did not attempt to recapitulate the crosstalk between the proximal tubule and peritubular capillaries, which is integral to kidney function. Furthermore, these early designs employed cells in a monolayer format, rather than the tubular architecture found in vivo.

**Figure 5:**
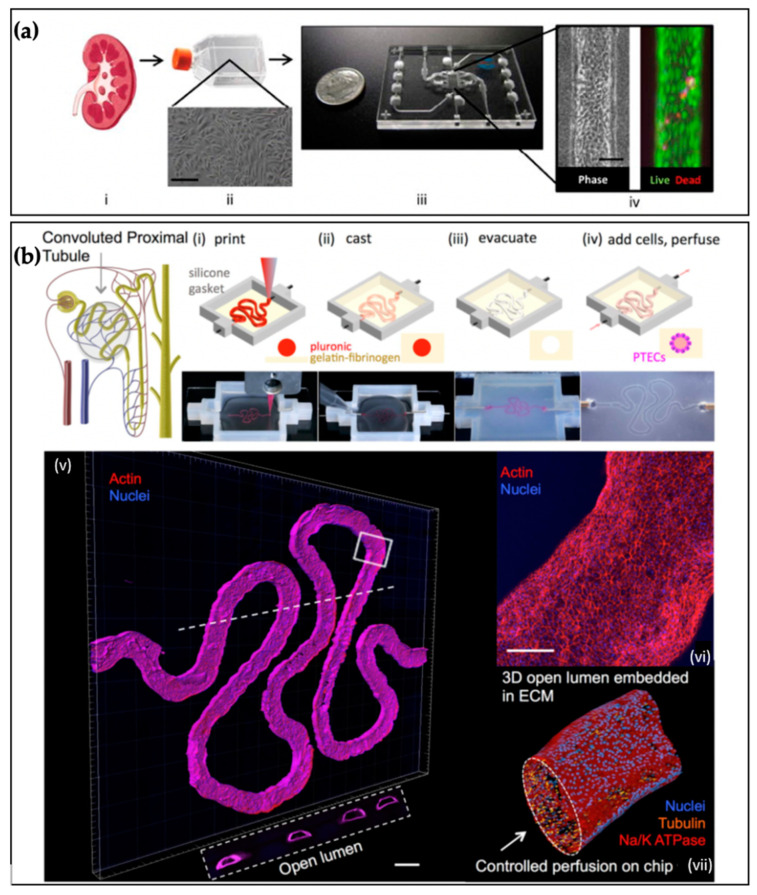
(**a**) Schematics of isolated human kidney tissue (**i**–**ii**) seeded into Nortis Inc.’s single-channel 3D MPS platform (**iii**) with phase contrast and live/dead images of primary RPTEpCs at day 28 (**iv**). Reproduced with permission from Elsevier [136]. (**b**) Convoluted proximal tubule schematic and images of Homan et al.’s 3D bioprinting fabrication steps (**i**–**iv**) and confocal 3D renderings of their RPTEpCs organized into a tubule with an open lumen: actin (red), nuclei (blue), and tubulin (orange). Reproduced with permission from [137]. The work was published under a CC BY license (Creative Commons Attribution 4.0 International License; https://creativecommons.org/licenses/by/4.0/).

Jang et al.’s model and other 2D planar systems allowed for investigations of well-differentiated TEpCs in environments where shear, stretch, and pressure can be finely tuned, but they lack critical 3D components of the nephron and kidney that could impact the accuracy and relevance of resulting data. These 3D characteristics include open tubule lumens lined on all sides by TEpCs, surrounding interstitial ECM and peritubular capillaries, and architecture consisting of convoluted and straight segments, amongst others. Additionally, when compared to 2D models, 3D kidney TCs have demonstrated cellular responses to nephrotoxins that more closely resemble those seen in vivo [134], and they also maintain the viability and differentiation of TEpCs for longer periods of time [135]. In 2016, Weber et al., using a platform developed by Nortis Inc., characterized a 3D flow-directed human renal proximal tubule microfluidic model (Figure 5a) [136]. Microfiber inserts were first positioned to maintain a hollow tubular structure, and then collagen I was injected and allowed to gel. Subsequently, the microfiber inserts were removed, and the device was flooded with collagen IV, a significant constituent of the RPTEpC basement membrane. After this luminal coating gelled, the model was seeded with primary human RPTEpCs, and media flow was initiated after 24 h. Researchers were able to generate a perfusable in vitro renal proximal tubule with dimensions comparable to its in vivo counterpart. The seeded RPTEpCs assumed appropriate polarity, demonstrating microvilli and the tight junction protein zonula occludens-1 at the apical aspects, and Na^+^/K^+^ ATPase and extensive membrane interdigitations at the basolateral aspects. RPTEpCs in this model did not express the kidney injury molecule-1 (KIM-1), an acute kidney injury marker that is expressed by kidney cells in 2D static culture. RPTEpCs in this model demonstrated glucose transport, ammoniagenesis, vitamin D bioactivation, and glutathione metabolic activity for over four weeks. The results suggested that 3D, flow-driven models of the proximal tubule offer advantages over 2D models beyond more accurate structural mimicry. In addition to recreating the proximal tubule, the device also had a “vascular” channel that, similar to the peritubular capillaries, could deliver solutes to the tubule’s exterior surface. By introducing para-aminohippurate and indoxyl sulfate into media flowing through this channel, the researchers demonstrated significant transport of these solutes through the collagen-filled “interstitial space” and into the tubule lumen. These renal TC advancements allow for renal tubular drug secretion studies that are not possible with traditional Transwell^®^ culture systems.

In 2016, Homan et al. were the first to develop a protocol that combined bioprinting and 3D cell culture to create 3D perfusable proximal tubules that were fully embedded within an ECM (Figure 5b) [137]. Bioprinting allowed the researchers to more accurately replicate the initial portion of the proximal tubule’s convoluted nature and generate tubule diameters of 150 to 700 μm. As in previous studies, the RPTEpCs subjected to fluid shear exhibited polarization, developed primary cilia and a robust microvilli brush border, and appropriately expressed Na^+^/K^+^ ATPase and AQP1. This TC could be used to qualitatively (via immunostaining) and quantitatively (via diffusional permeability measurements) assess acute and chronic drug-induced nephrotoxicity due to the 65-day-plus viability of the incorporated cells. In 2019, some of the same researchers expanded on this model by incorporating a peritubular capillary adjacent to the proximal tubule, with a thin layer of permeable ECM separating the two tubular structures. This 3D VasPT, as the researchers called it, was housed within a closed-loop perfusion system and used to investigate the renal reabsorption of albumin and glucose, as well as the effects of hyperglycemia [138]. The 3D VasPTs recapitulated the selective reabsorption capacity of RPTEpCs and the peritubular capillary-proximal tubule crosstalk seen in vivo. However, the tissue models were only around 20% as efficient as in vivo counterparts regarding glucose reabsorption. Therefore, while this TC made substantial improvements to the 2016 model, opportunities exist to further enhance the reabsorption and secretion capabilities of in vitro proximal tubules.

Rein et al., including researchers who worked on these bioprinted proximal tubule TCs, in 2020 created an in vitro model of the cortical collecting ducts (CCDs) [139], which contribute to the maintenance of the total body electrolyte, acid/base, and fluid homeostasis, and exert significant control over blood pressure [140,141,142,143]. This group of researchers used 3D printing to generate the TC’s frame and provided a tubule lumen by inserting a 510 μm diameter pin through holes in opposite edges of the frame before filling the chamber with a gelatin/fibrin ECM. After allowing the ECM to solidify, the pin was removed, and the lumen seeded with immortalized mouse mpkCCD cells. This cell line exhibits phenotypes similar to principal cells (PCs), having the capacity to reabsorb Na^+^ and water and secrete K^+^. Within one week of initiating media perfusion, the researchers observed a tight epithelial barrier composed of differentiated and polarized PCs displaying apical epithelial Na^+^ channels (ENaCs) and basolateral Na^+^/K^+^ ATPases. This model has the potential to explore the molecular mechanisms responsible for the regulation of transport within CCDs.

Until recently, glomerulus TCs have had limited success because immortalized podocytes readily dedifferentiate when cultured in vitro. In 2017, Musah et al. significantly advanced the field of kidney TC engineering by demonstrating an efficient (>90%) and chemically-defined protocol to differentiate hiPSCs into podocytes [144]. These researchers were able to show that the hiPSC-derived podocytes expressed markers of the mature phenotype (nephrin^+^, WT1^+^, podocin^+^, Pax-2^−^) and exhibited primary and secondary pedicles, which are largely absent in immortalized podocyte cell lines. The researchers then adapted this protocol to differentiate hiPSCs into podocytes within a microfluidic device. The hiPSCs were seeded into one channel of a microfluidic device separated by a permeable membrane (mimicking the glomerular basement membrane) from another microfluidic channel coated with human glomerular ECs. Podocyte differentiation media was perfused through the hiPSC channel, and EC culture medium flowed through the other, and cyclic strain (mimicking that generated in the glomeruli with each cardiac cycle) was applied. Compared to podocytes differentiated under fluid flow alone, these hiPSC-derived podocytes demonstrated more intense nephrin staining and an increased cytoplasmic to nuclear nephrin staining pattern, indicating a more mature phenotype. The hiPSC-derived podocytes produced collagen IV within this glomerular TC, mimicked podocyte-capillary wall interactions, and demonstrated a differential filtration of albumin and inulin.

### 5.6. Limitations

Over the past decade, there have been significant advancements within the field of kidney TCs. However, the kidney remains one of the most challenging organs to fully reconstruct in vitro because it comprises 26 cell types and is organized into such intricate functional units [145]. While current efforts mimic specific aspects of kidney function, such as glomerular filtration or tubular reabsorption, there has been little to no progress towards a single platform that combines these complex functions. Current models also do not accurately replicate fluid flow within different parts of the nephron. Fluid flow is associated with specific hemodynamic stresses, such as pressure, shear, and stretch, which affect cellular organization and function and could be critical in replicating in vivo-like tissue behavior. The greater characterization (and further development) of immortalized kidney cell lines, a more detailed understanding of renal tubular reabsorption and secretion functions, and the continued incorporation of associated vasculature will hopefully provide low-cost and more accurate alternatives to the current methods employed in therapeutic studies and disease modeling.

## 6. Microphysiological Systems

While recent advances in TC engineering have allowed for more refined and reproducible approaches to tissue-/organ-specific drug toxicity screening, bridging the gap between pre-clinical studies and human trials without animal models requires recreating critical system-level interactions between interdependent organs and tissues with microphysiological systems (MPSs). Incorporating these multisystem interactions is necessary because drug toxicity is frequently not limited to a single organ or tissue. For instance, many patients receiving cancer treatment develop chemotherapy-induced cardiotoxicity (CIC), even when receiving targeted therapeutics [146]. While agents targeted at preventing CIC, such as dexrazoxane, exist, they frequently decrease the chemotherapy’s effectiveness. MPSs that recreate interactions between the tumor microenvironment and cardiac tissue could be used to screen combinations of chemotherapeutics and cardioprotective therapies to evaluate both tumor regression and cardiotoxicity. More generally, in addition to the target organ/tissue, it may be beneficial to incorporate elements of the gastrointestinal (GI) system or skin to evaluate drug routes of entry; the circulatory system, to model the distribution of the drug; the liver, to understand metabolic breakdown of a prodrug or drug; and the kidneys, to determine the rate of clearance of the drug from the body. As with drug screening applications, TCs allow for the more precise modeling of tissue-/organ-specific disease processes than animal models, but their accuracy is limited by the lack of functional interactions between interdependent organ systems. One example of the type of multisystem disease process not captured by TCs is the pathophysiology of secondary hyperparathyroidism (SHPT). SHPT frequently develops due to deformations in vitamin D metabolism and calcium-handling in dysfunctional kidneys, and the resultant abnormal elevation of parathyroid hormone leads to increased levels of intracellular calcium and oxidative stress, provoking pathological cardiac remodeling, conduction abnormalities, coronary artery and heart valve calcification, and hypertension [147]. Similarly, while TCs can incorporate immune cells, they lack lymphoid tissues, such as bone marrow, spleen, and lymph nodes, without which the activation, expansion, and recruitment of immune cells to different parts of the body in response to injury or infection cannot be modeled.

**Figure 6:**
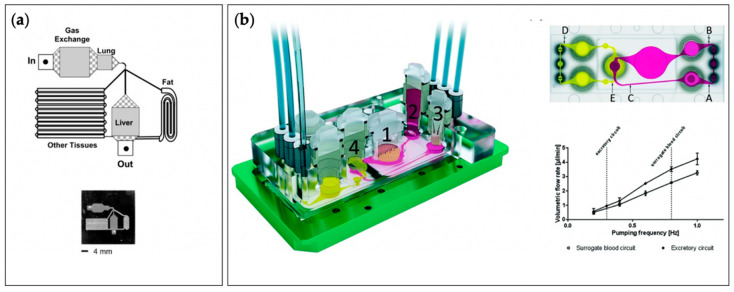
(**a**) The first example of a multi-organ tissue chip designed by Viravaidya et al. to recreate interactions between the liver, adipose tissue, and the lung to probe naphthalene toxicity. Reproduced with permission from John Wiley and Sons [148]; (**b**) A complex four-organ tissue chip developed by Maschmeyer et al. where interactions between the intestine (1), liver (2), skin (3), and kidney (4) were recreated, and circulatory and excretory circuits established. Reproduced with permission from the Royal Chemical Society [154].

One of the earliest MPS platforms for drug toxicity studies was the microscale cell culture analog developed by Viravaidya et al. in 2004 (Figure 6a) [148]. This platform was designed to recreate interactions between the lungs and liver to model naphthalene metabolism and incorporated cell-free microfluidic channels of differing geometries to recapitulate fluid distribution dynamics in tissues perfused at different rates. Since then, there have been multiple other examples of successful MPSs that integrate two or more tissue compartments. Several groups have developed heart-liver MPSs to study liver-metabolized drug effects on cardiomyocyte function [149,150]. An example of a heart-liver-vascular MPS is the HeLiVa platform developed by Vunjak-Novakovic et al. in 2013, in which liver and cardiac micro-“tissues” were functionally connected by EC-lined vascular channels, with all incorporated cells having been derived from iPSCs [151]. These cells’ origins are critical, as models incorporating iPSC-derived cells are suitable for patient-specific drug screening applications. In 2017, Skardal et al. published a model that successfully incorporated heart, lung, and liver TCs together in a closed circulatory system [152]. The researchers observed in vivo-like drug responses from cells in this MPS, illustrating the value of integrating multiple TCs for drug testing. In 2020, Schimek et al. developed a lung-liver MPS to model the effects of inhaled substances metabolized by the liver, enabling the assessment of both toxicity and the bioavailability of respiratory agents [153]. Using this model, the researchers saw differing responses of bronchial epithelial cells exposed to aflatoxin B_1_ when they were cultured with HCs in a separate chamber versus when the liver cells were excluded. This demonstrated that the device enabled crosstalk between the two cell populations analogous to that seen in vivo. This platform could become a powerful tool for pharmaceutical development and personalized medicine. Lastly, in 2015, Maschmeyer et al. developed a four-organ-chip MPS that connected the human intestine, liver, skin, and kidney and provided physiologic fluid flow to each distinct “organ” (Figure 6b) [154]. Using this model, the researchers demonstrated the establishment of homeostasis between the organ chambers within 2 to 4 days. They observed cell viability and the barrier integrity of the kidney and intestine components for over 28 days, making the four-organ-chip MPS a suitable platform for absorption, distribution, metabolism, and excretion drug profiling studies, as well as repeated dose, long-term drug toxicity screens. For further reading on MPSs, please see Table 1.

## 7. Challenges Associated with Design and Construction of Microphysiological Systems

### 7.1. Communication

The primary challenge associated with integrating multiple TCs to form an MPS is establishing communication between two or more “tissues” or “organs” that accurately recreates the crosstalk experienced in vivo. As seen above, technologies, such as photolithography, soft lithography, micropatterning, 3D printing, bioprinting, and microfluidics, can be used to fabricate co-culture models that mimic in vivo tissue organization and cell–cell/cell–ECM contacts. This allows for direct communication between different “tissues” or “organs” and recapitulates critical parameters influencing mechanotransduction signaling pathways. Communication among tissues/organs that are not in direct contact with one another can be enabled. Devices engineered to house different tissue/organ models within separate chamber that are connected via microfluidic channels ensure soluble factor signaling via culture medium circulation. Microfluidic devices can also be adapted to facilitate more complex signaling in several ways, such as utilizing different microfluidic channel architectural features and micro/nanoscale pore structures to establish spatiotemporal molecular gradients [172,173] and particle size exclusion [174,175]. Functional physiologic barriers can also be generated by culturing appropriate cell types in architectures that mimic in vivo organization.

In vitro culture of cells and tissues involves specific cell culture media formulations to maintain viability and in vivo-like phenotype and function [176]. In the human body, blood plays the role of the cell culture medium with supporting tissues and cells producing the necessary growth factors. MPSs frequently do not include all of the supporting cells necessary to provide the specific soluble factors for maintaining the viability, phenotypes, and functions of the incorporated “tissues”/”organs.” This is often by choice, as researchers are only seeking to model specific tissues and organs and want to minimize extraneous variables. However, in most cases, it is also difficult to incorporate all the requisite supporting cells (which differ based on the “tissue”/”organ” composition of the device). Thus, considerations must be made concerning what media formulations are necessary for various combinations of “tissues”/”organs.” To simplify device operation and simulate how different tissues and organs communicate in vivo, researchers frequently seek to use one medium supplemented with the various nutrients and soluble factors necessary to satisfy the incorporated “tissues”/”organs”. Formulating this sort of common medium can be very challenging, as specific soluble factors required by one cell type can negatively impact the function or phenotypic maintenance of another.

The manner in which the medium is perfused through MPSs is another important parameter to consider. Establishing and maintaining physiological fluid flow among multiple “tissues”/”organs” is challenging, as in vivo flow rates vary significantly in different tissues/organs. As discussed previously, fluid flow is associated with shear stress, pressure, and stretch. In vivo, most tissues/organs have extensive capillary networks through which blood flows very slowly, exposing ECs lining the microvessels to low shear stress and pressure and the surrounding cells of the tissues/organs to negligible stretch. Microfluidic technology allows for channels within MPSs that provide fluid flow within the main perfusion networks but do not directly interact with cells comprising the “tissues”/”organs,” thus utilizing primarily diffusive transport to deliver oxygen and nutrients to the cells [177]. However, one caveat to this approach is that microvascular networks or other small lumens must be established within the “tissues”/”organs” if their thicknesses are scaled up beyond a couple of hundred μm, due to the diffusion limitation of oxygen [91,92]. Arterial and venous vascular tissues, unlike capillaries, are subject to non-negligible amounts of shear stress. As discussed previously, combinations of lithographic techniques and 3D printing can fabricate vascular channels that mimic the geometry of in vivo vasculature. By tuning the stiffness of whatever material surrounds these vascular channels and employing physiologic flow profiles, one can reproduce physiologic shear stress, pressure, and stretch on the ECs lining the lumens. Microfluidic systems are compatible with various active and passive pumping techniques that can generate steady and pulsatile flow. The heart is a pulsatile pump and contains four valves that ensure blood moves in the forward direction. Likewise, in vitro, unidirectional flow control valves must be incorporated to ensure that the cyclic filling and contraction of the “heart” propels cell culture medium in the intended direction. Using microfluidic approaches, several groups have developed both active and passive flow control valves that can be used to ensure pulsatile pumping and unidirectional flow, similar to that seen in the body [178,179,180]. Microfabrication techniques can also create alternatives to cardiac tissue via the development of pumps to generate pulsatile flow, similar to the heart [35,181]. In summary, fluidic circuits can be tailored via adjustments in geometry, perfusion rate, and organization, along with incorporating components, such as actuators and valves (both in tubing connecting different TCs and within the TC devices themselves), to meet perfusion and transport requirements.

### 7.2. Nondestructive Monitoring

MPS platforms are functionally more complex and more challenging to construct than individual TCs. Therefore, the efficient utilization of these complex multi-organ MPSs is best accomplished if the monitoring of cellular/”tissue”/”organ” function can be performed in a non-destructive fashion, thereby enabling periodic or even continuous measurements of cellular responses. Non-destructive testing allows multiple drugs or therapeutic strategies to be tested with the same platform in a sequential fashion. Soft lithography microfabrication techniques are often employed to fabricate the structural housing for tissue culture, whereas conventional surface and bulk micromachining techniques, frequently used for semiconductor fabrication, have been utilized to enable on-chip biosensing. Biosensing includes the measurement of biophysical signals, such as cellular action potentials, intracellular calcium signaling, transepithelial electrical resistance (TEER), and cellular impedance [182], as well as biochemical measurements, including gene expression, intracellular and secreted proteins, biomarkers of injury, and measurements of local pH and oxygen levels. Several devices enable the non-destructive measurement of cellular action potentials via microelectrode arrays or nanoneedle arrays [183,184,185]. Additionally, endothelial and epithelial TCs have been fabricated with micro/nanoporous membranes coupled with electrodes to allow for non-destructive TEER measurements [186,187]. Micromachined electrodes have also been used to measure cellular electrical resistance and impedance, which provide information regarding cell size and intracellular complexity [152,188]. Sensors have been developed to measure local oxygen, pH, and levels of signaling molecules, such as nitric oxide [84,189,190]. Antibody arrays have been integrated downstream of cell cultures to measure soluble factor production, where the quantitative assessment of specific molecules is converted to optical or electrical signals that can be continuously monitored [191]. Overall, while there have been some critical technical advances enabling non-destructive readouts from MPSs, integrating simple and reliable readouts that can be used to evaluate changes that occur as a consequence of toxicity or disease is essential. Continued progress in biosensing will hopefully significantly increase the number of assayable signals and the sensitivity and specificity with which they can be measured. Such advancements will be critical to the widespread acceptance and usage of complex multi-cellular MPS in the greater scientific community.

### 7.3. Material Selection and Fabrication of TCs and MPS

The selection of materials and fabrication techniques used to build TCs and MPSs requires careful consideration of the tissue/organ model of interest, culture conditions, desired electrical and mechanical stimuli, throughput of the system, biocompatibility issues, and methods used for monitoring and detection. TC and MPS platforms require biocompatible materials to provide structural support for engineered tissues and to promote healthy or diseased tissue phenotype and function. Desirable biomaterial properties include minimal cellular toxicity [192], minimal absorption of biomolecules and drugs [189], transparency to facilitate on-chip imaging, and sufficient gas permeability to ensure oxygen transport to cells in culture. PDMS fulfills many of these requirements and is widely used in the early-stage development and prototyping of TCs and MPSs [190]. More complex or advanced models may employ other polymers such as polycarbonate, polystyrene, and polymethyl methacrylate. These compounds are compatible with high-throughput fabrication techniques such as injection molding, embossing, and 3D printing. Glass is also frequently used in the construction of TCs and MPSs as it fulfills many of the requirements for supporting cell/tissue culture and is compatible with the integration of active sensing and actuating elements. TCs and MPSs that require the integration of electrodes and electrochemical sensors have also utilized semiconductor materials, such as silicon, for their construction. The main drawback of glass and silicon is that complex fabrication techniques are necessary, and silicon is not an optically transparent material.

## 8. Summary

Overall, TCs and MPSs have great potential to revolutionize drug discovery, drug toxicity testing, and disease modeling by providing models of human health and disease. There has been significant progress in ensuring that TCs accurately replicate human physiology and in the engineering of complex interactions between different TCs. Despite this progress, TCs and MPSs have yet to find widespread application in establishing disease models and discovering and evaluating drugs. This can be attributed to limitations in the ability of TCs to completely replicate the in vivo environment and complexities associated with the construction and operation of TCs and MPSs. Microfluidics provides unique opportunities to address these issues and enable the design of a modular plug and play TCs that accurately mimic critical aspects of the in vivo environment in a simple and easy-to-use manner. Finally, we stress that, with advances in the complexity of engineered systems, it is also important to ensure that the developed platforms are also simple to use, inexpensive, and highly reproducible to ensure widespread adoption in research and industry.

## Figures and Tables

**Table 1:** Microphysiological Systems.

Year	Author	Organs/Tissues	Application
2004	Viravaidya et al. [148]	Lung–Liver–Fat	Napthalene toxicity
2013	Wagner et al. [155]	Liver–Skin	Drug Testing
2013	Vunjak-Novakovic et al. [151]	Heart–Liver–Vascular	Drug Testing
2014	Lin et al. [156]	Bone–Cartilage	Drug Testing
2014	Clark et al. [157]	Liver–Tumor	Testing Therapeutic Strategies
2015	Maschmayer et al. [158]	Liver–Skin/Intestine	Drug Testing
2015	Maschmayer et al. [154]	Intestine–Liver–Skin–Kidney	Drug Pharmacodynamics and Toxicity
2016	Esch et al. [159]	GI Tract–Liver	Disease Modeling
2016	Moura Rosa et al. [160]	Lymph Node–Immune Cell	Disease Modeling and Drug Testing
2017	Loskill et al. [161]	Adipose–Vascular	Disease Modeling
2017	Skardal et al. [152]	Liver–Heart–Lung	Drug Efficacy and Toxicity
2017	Tsamandouras et al. [162]	Gut–Liver	Drug Pharmacokinetics
2018	Oleaga et al. [150]	Heart–Liver	Cardiotoxicity
2019	McAleer et al. [149]	Heart–Liver	Terfenadine Pharmacokinetics
2020	Yin et al. [163]	Heart–Liver	Testing Anti-depressant Drugs
2020	Schimek et al. [153]	Lung–Liver	Toxicity of inhaled substances
2020	Baert et al. [164]	Liver–Testis	Reproductive toxicity
2020	de Mello et al. [165]	Heart–Liver–Skin	Topical Drug Delivery
2020	Kwak et al. [166]	Skin–Vasculature	Immune responses
2020	Sung et al. [167]	Gut–Liver	Drug Testing
2020	Clark et al. [168]	Liver–Tumor	Tumor Metastasis
2020	Jeon et al. [169]	Gut–Liver–Immune Cell	Modeling inflammatory responses
2020	Marin et al. [170]	Liver–Intestine	Drug pharmacological and toxicological assessment
2021	Giordano et al. [171]	Gut–Kidney	Chronic Kidney Disease Modeling
